# A random regression model for total litter weight in mice

**DOI:** 10.1093/jas/skag060

**Published:** 2026-02-23

**Authors:** Ricarda Elisabeth Jahnel, Martina Langhammer, Norbert Reinsch

**Affiliations:** Research Institute for Farm Animal Biology (FBN), Dummerstorf 18196, Germany; Research Institute for Farm Animal Biology (FBN), Dummerstorf 18196, Germany; Research Institute for Farm Animal Biology (FBN), Dummerstorf 18196, Germany

**Keywords:** litter traits, birth weight, maternal genetic effects, direct genetic effects, multiparous mammals

## Abstract

Measuring litter weight (LW) as a summary trait in multiparous animal species is less cost-intensive than individual birth weight. We propose a random regression model for total LW, where the genetic effects of dams and sires are modeled as linear random regression slopes on litter size with potentially different variances. Restricted maximum likelihood estimation of variance components was conducted separately for four diverse mouse lines, and the results were compared. In total, there were 11,430 total LWs, representing 175,446 pups. The sire genetic variance component was significant in two lines. The random regression model implies that the heritability of the trait and the reliability of estimated genetic effects depend on litter size, in contrast to previously used models. When averaged over different litter sizes, heritability for total LW was moderate to low at 0.32 in the unselected control line FZTDU, at 0.19 in the growth-selected line DU6, and at 0.14 and 0.12 in the high-fecundity lines DUC and DUK, respectively. In conclusion, the proposed random regression model makes more detailed use of the information available in total LW data. It provides potential applications in other multiparous species, for a cost-effective selection for birth weight using LW data.

## Teaser

A random regression model for total litter weight is proposed, which is in accord with animal models with direct and maternal genetic effects. It offers new opportunities for a cost-effective selection for birth weight in multiparous farm animals.

## Graphical abstract

**Graphical Abstract:**
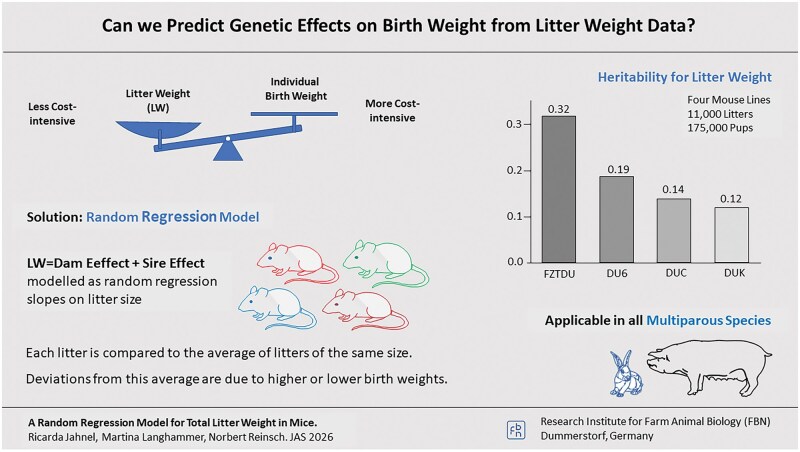

## Introduction

The genetics of birth weight has been studied in multiparous species such as mice (Eisen 1978; Langhammer et al. 1987; Ogawa and Satoh 2020), pigs (Estany and Sorensen 1995; Damgaard et al. 2003; Roehe et al. 2010; Zaalberg et al. 2023), minks (Karimi et al. 2018), and rabbits (Argente et al. 1999; Sorensen et al. 2001; Ezzeroug et al. 2020). Hereby, the litter weight (LW) of animals can be either taken at once as a single weight measurement (Gutiérrez et al. 2006; Varona et al. 2007) or by adding a series of individual birth weights, taken separately for every single newborn (Formoso-Rafferty et al. 2017). Individually measured birth weight provides more details beyond LW, but recording it is labor and cost-intensive, compared to total LW. Three major components contribute to the genetic variation of LW. First, LW increases with litter size (LS), which depends on the mother’s genotype, controlling ovulation rate, and uterine capacity (Eisen and Durrant 1980; Luxford and Beilharz 1990). The newborns’ prenatal growth genotype makes up the second component. And third, there is the maternal genetic effect of the mother on the growth of her offspring.

This maternal effect has been of primary interest in research scenarios, where litters were first standardized to a predefined size after birth, and LW was later measured at weaning (Falconer 1955; Eisen and Durrant 1980). This particular design avoids the confounding between LW and LS (Eisen 1978) and allows to interpret LW as an indicator of mainly maternal effects on pre-weaning growth. Consequently, in this setting, LW has been considered a trait of the mother. Newborn litters, however, have variable LS, and for them, there is no available experimental design that avoids the confounding of LW with LS. This confounding has been treated in different ways in previous publications on the LW of newborns, which all stuck to the concept of LW as an exclusively maternal trait. Some authors have made adjustments for LS—either by regressing on the number of newborns (LWA; Chimonyo et al. 2006), or by calculating average birth weight (ABW) from LW and the number of individuals (Schüler et al. 1990). Still, other authors (Luxford and Beilharz 1990; Sell-Kubiak 2021) have analyzed total LW as a maternal trait without any further adjustment for LS (TLW). Furthermore, to the best of our knowledge, previous studies have not included any type of genetic effect in the model that would explain the average direct genetic effect of litter mates on their own birth weight and thus LW.

Our study improves on previous practice by introducing a random regression model for LW (RRM). This model integrates the genetic effects of both parents on ABW and accounts for the variable number of pups by defining parental genetic effects as random linear regression slopes on LS. We explain in detail how this model reflects both the maternal genetic effect of the dam and the average direct genetic effects of both parents on the average prenatal growth of littermates. Also, it automatically accounts for the variable amount of information provided by litters of different sizes. As a use case, corresponding genetic parameters were estimated separately for four diverse mouse lines, and the results were compared. Analyses were based on a total of 11,430 LWs, representing 175,446 pups.

## Material and methods

### Mouse lines and data

Mice were kept under a general permission for breeding and maintaining laboratory mice, issued by the district veterinary authority on 7 November 1991. All data were routinely measured by weighing and counting animals, with no further intervention. Federation of European Laboratory Animal Science Associations (FELASA) guidelines for animal care and husbandry were always followed.

LW data were sourced from four different mouse lines, which had been developed at the Research Institute for Farm Animal Biology’s (FBN) Lab Animal Facility over an extended period (Dietl et al. 2004). The first line is the unselected control line FZTDU, which originally was established by crossing eight different founder lines. Four of these strains were outbred, and the other four were inbred (Schüler 1985; Dietl et al. 2004). From the beginning, a randomized mating scheme at a population size of 125 breeding pairs with a 1:1 mating ratio was used to avoid inbreeding. The average mating age of mothers was 69.4 ± 3.9 d. The other three lines were later branched off from the FZTDU population with 60 to 80 breeding pairs per generation. As in FZTDU, females and males, if chosen for a mating, had only a single opportunity to reproduce. There are a few exceptions only in DU6, where a limited number of males were mated to two females. The DU6 line has been selected for high body weight at d 42 since 1975. Most of the time, family selection (Renne et al. 2003) was exerted by ranking litters according to the body weight of two randomly chosen males. It was only in later generations that all offspring of a litter got a phenotypic record for body mass, and selection between individuals was on estimated breeding values from a best linear unbiased prediction (BLUP) animal model. As a result of the selection, the DU6 line stands out from the other lines by its large average body weight, which is more than 2-fold that of the control line. Family selection was also applied in the high fecundity lines DUK and DUC during most of their selection history. Litters were first ranked according to an index combining LS and LW and then parents of the next generation were randomly chosen from about the top half of all full-sib families. Later selection was on estimated breeding values for LS, with no reference made anymore to LW. All four populations underwent a severe bottleneck in population size when only a limited number of litters were brought to a specified pathogen-free environment in a new mouse facility via embryo transfer in 2010. The number of generations that had already passed before that transfer was 164 in FZTDU, 154 in DU6, 162 in DUC, and 164 in DUK. It was from then on that selection was based on estimated breeding values. Analyzed data and pedigrees were restricted to generations kept under specified pathogen-free conditions in the new mouse facility. Therefore, all estimates of genetic variances represent genetic variation at the beginning of that period.

**Figure 1:**
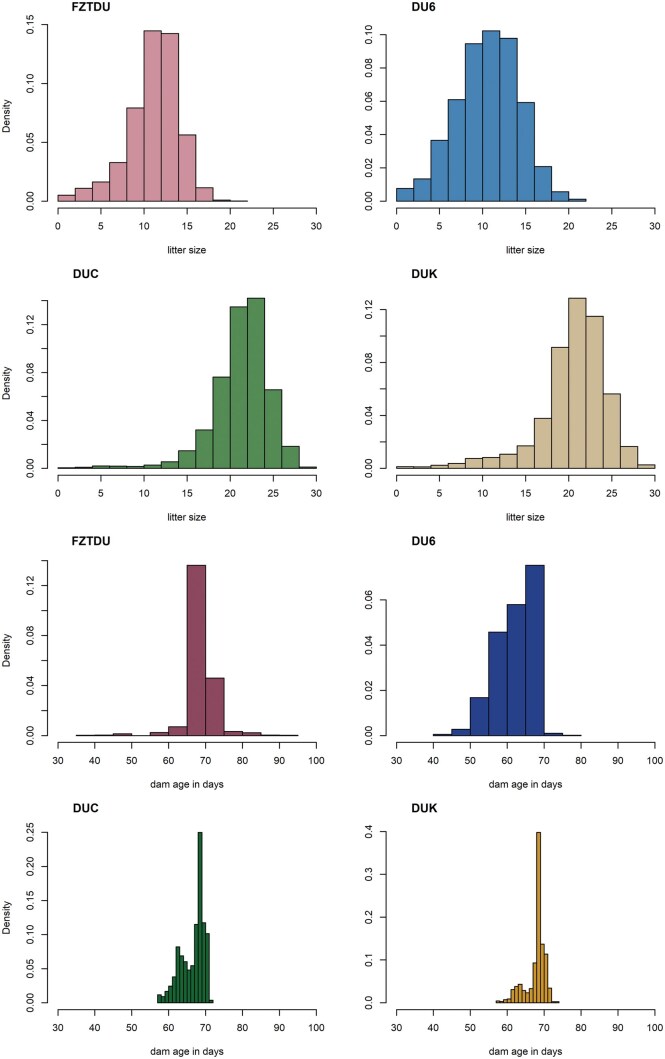
The upper four panels display the distributions of litter size in the lines FZTDU, DU6, DUC, and DUK (clockwise). The following four panels depict the distributions of dam age at birth (in days) in the same order.

Table 1 provides structural information on the analyzed LW data from all mouse lines, while Figure 1 displays the distributions of LS and the age of the dam at birth. The number of litters exceeded two thousand in the long-term selection lines DU6, DUC, and DUK, and was more than double that in the control line, which had a higher population size. The total number of pups well exceeded 57,000 in FZTDU, due to numerous litters, and was around 48,000 in DUC and DUK, a consequence of the large average LS in the high fecundity lines. The number of pups was lowest at just over 22,000 in DU6, where the number of litters was similar to DUC and DUK, but the average LS was comparable to that of control mice (Table 2). More than 40 distinct generations from each line were included in the analyses. Total pedigree size exceeded 24,000 individuals in the larger control line FZTDU, a number that was approximately 10,000 fewer in the other lines.

**Table 1:** Structural characteristics of the analyzed litter weight data from mouse lines FZTDU, DU6, DUC, and DUC.

Parameter	Mouse line			
	FZTDU	DU6	DUC	DUK
**Pups (no.)**	57,012	22,391	48,056	47,987
**Litters (no.)**	4,909	2,025	2,210	2,286
**Generations (no.)**	42	44	42	42
**Pedigree size (no.)**	24,315	15,748	12,007	12,577

**Table 2:** Descriptive statistics for litter traits of the mouse lines FZTDU, DU6, DUC, and DUC.

Trait		Mouse line			
	Parameter	FZTDU	DU6	DUC	DUK
**LS (pups)**	Mean	11.61	11.06	21.74	20.99
	SD	2.98	3.66	3.38	4.08
	Range	1–22	1-22	1–30	1–30
**LW (g)**	Mean	20.65	28.6	39.78	37.31
	SD	4.79	9.02	5.63	6.08
	Range	1.19–43.90	2.14–57.04	1.87–53.78	1.98–51.28
**ABW (g)**	Mean	1.81	2.62	1.84	1.80
	SD	0.20	0.28	0.13	1.17
	Range	1.15–3.14	1.54–5.56	1.65–2.66	1.17–2.99

Abbreviations: LW, litter weight; LS, litter size; ABW, average birth weight per litter; Mean, sample average.

In DU6, 236 bucks of a total of 1,789 bucks were the fathers to multiple litters; on average, the number of litters per buck was 1.32 ± 0.34. In contrast, in all other lines, there was only a single litter per buck.

All litters were born between November 2011 and October 2022. Within the first day after birth, LW was measured by jointly putting all living male and female pups on a scale (type “Cubis MSA”, Sartorius Lab Instruments GmbH & Co. KG, Germany; Table 1). Any visible stillborn pups were not added to the LS and not weighed, as cannibalism is known to mask early pup mortality in mice. Animals were kept in 267 × 207 × 140 mm polysulfone cages (H-Temp PSU, Type II, Eurostandard, Tecniplast, Germany) or Type II long cages (365 × 207 × 140 mm), where *ad libitum* water and food (standard breeding diet; ssniff M-Z autoclavable, ssniff Spezialdiäten GmbH Soest, Germany) were available. Housing temperature was 22.5 °C, humidity was kept above 50%, and there was a controlled 12:12-h dark-light cycle.

### Random regression model

Total LW was measured by jointly putting all living pups of a newborn litter on a scale. According to this practice, individual birth weights remained unknown, and any technical measurement error or random biological deviation jointly affected the outcome at the group level (litter), resulting in a single residual$e_{i}$owned by all littermates.

Concerning genetic effects on individual birth weight, there are plenty of studies in multiparous species such as mice and pigs, which have demonstrated the importance of both maternal and direct genetic effects (e.g. Roehe et al. 2010; Zaalberg et al. 2023). Considering these random genetic effects determining the individual birth weights $y_{1}$ and $y_{2}$ of two litter mates, we have the following equations:


$$\left[\begin{array}{c} y_{1}\\ y_{2} \end{array}\right]=\left[\begin{array}{c} m_{d}\\ m_{d} \end{array}\right]+\left[\begin{array}{c} ta_{d}\\ ta_{d} \end{array}\right]+\left[\begin{array}{c} ta_{s}\\ ta_{s} \end{array}\right]+\left[\begin{array}{c} r_{1}\\ r_{2} \end{array}\right],$$

where $m_{d}$ is the maternal effect of the dam of the litter, and $ta_{d}$ and $ta_{s}$ are half of the dam’s and sire’s direct breeding value for individual birth weight, and $r_{1}$ and $r_{2}$ are residuals, accounting for measurement error, other non-genetic variation, and the direct Mendelian sampling term of each individual.

The sum of $y_{1}$ and $y_{2}$, exemplifying the weight of a small litter of size two, then is given by


$$y_{1}+y_{2}=2m_{d}+2ta_{d}+2ta_{s}+e_{i},$$

where the sum $r_{1}+r_{2}$ is replaced by a single joint residual $e_{i}$ for litter *i.* Now, the sum of the two individual Mendelian sampling effects in $r_{1}$ and $r_{2}$ contributes to this joint residual. It should be noted, that with variable LS this contribution is a potential source of heteroscedastic residual variances. However, in the following, we assume that the residual variance is not affected by LS. Generalizing to any given LS$n_{i}$, we get the following equation for the LW $y_{i}$:


$$y_{i}=n_{i}(m_{d}+ta_{d}+ta_{s})+e_{i}.$$

Now the overall genetic effect of the dam can be seen as a linear random regression slope $a_{d}=(m_{d}+ta_{d})$ on the number of pups. In analogy, the genetic sire effect has a linear random slope $a_{s}=ta_{s}$.

Changing perspective from litters to animals, we consider a base animal and its maternal and direct breeding valuesmandbfor individual birth weight, as enshrined in its DNA. Therefrom we derive two additive effects on LW: the base animal’s dam effect ad=(m+12b) and its sire effect as=12b. For a particular LS, these effects have to be multiplied by LS. Note that for the sake of simplicity, the indices *d* and *s* now denote the two different genetic effects of the same animal. In matrix notation, this can be summarized as


$$\left[\begin{array}{c} a_{d}\\ a_{s} \end{array}\right]=\left[\begin{array}{cc} 1 & \frac{1}{2}\\ 0 & \frac{1}{2} \end{array}\right]\left[\begin{array}{c} m\\ d \end{array}\right]=K^{\prime }\left[\begin{array}{c} m\\ d \end{array}\right].$$

The (co)variance of the genetic effects in the random regression model G then is


$$G=\mathrm{Var}\left[\begin{array}{c} a_{d}\\ a_{s} \end{array}\right]=\left[\begin{array}{cc} \sigma_{t}^{2} & \sigma_{t,s}\\ \sigma_{t,s} & \sigma_{s}^{2} \end{array}\right]=K^{\prime }\left[\begin{array}{cc} \sigma_{m}^{2} & \sigma_{m,d}\\ \sigma_{m,d} & \sigma_{d}^{2} \end{array}\right]K=\left[\begin{array}{cc} \sigma_{m}^{2}+\frac{1}{4}\sigma_{d}^{2}+\sigma_{m,d} & \frac{1}{2}\sigma_{m,d}+\frac{1}{4}\sigma_{d}^{2}\\ \frac{1}{2}\sigma_{m,d}+\frac{1}{4}\sigma_{d}^{2} & \frac{1}{4}\sigma_{d}^{2} \end{array}\right],$$

where the variances and the covariances of the genetic random slope effects of dams and sires have an interpretation in terms of the direct and maternal genetic (co-)variance components $\sigma_{d}^{2},\;\sigma_{m}^{2}$, and $\sigma_{m,d}$ (additive-genetic variances of the direct and maternal genetic effects and their covariance). Once estimates of the three (co)variance components $\sigma_{t}^{2},\;\sigma_{s}^{2}$, and $\sigma_{t,s}$ have been obtained, they can be transformed into estimates of $\sigma_{d}^{2},\;\sigma_{m}^{2}$, and $\sigma_{m,d}$− by reversing the latter equation. Likewise, as $K^{\prime }$ is invertible, estimates of $a_{d}$ and $a_{s}$ can be converted into estimates of m and d.

The genetic variance of an observation with LS $n_{i}$ equals the variance of the sum of the parental effects $\sigma_{a}^{2}=\mathrm{Var}(n_{i}a_{d}+n_{i}a_{s})=n_{i}^{2}\sigma_{t}^{2}+n_{i}^{2}\sigma_{s}^{2}$ in the base population, and the phenotypic variance is $\sigma_{p}^{2}=\sigma_{a}^{2}+\sigma_{e}^{2}$, assuming that the residual variance does not change with LS.

### Heritability

The regression $h_{\mathrm{dam}}^{2}$ of the genetic dam effect of a litter’s mother on the LW phenotype (dam heritability) is $h_{\mathrm{dam}}^{2}=\frac{n_{i}^{2}\sigma_{t}^{2}}{n_{i}^{2}(\sigma_{t}^{2}+\sigma_{s}^{2})+\sigma_{e}^{2}}$. In analogy, the regression $h_{\mathrm{sire}}^{2}$ of the genetic sire effect of a litter’s father on the LW phenotype (sire heritability) is $h_{\mathrm{sire}}^{2}=\frac{n_{i}^{2}\sigma_{s}^{2}}{n_{i}^{2}(\sigma_{t}^{2}+\sigma_{s}^{2})+\sigma_{e}^{2}}$.

Both kinds of heritability vary with LS, reflecting the fact that a litter with more offspring provides more information on a parent’s genetic effect.

The total heritability of the trait, therefore, is


$$h_{t}^{2}=\frac{n_{i}^{2}(\sigma_{t}^{2}+\sigma_{s}^{2})}{n_{i}^{2}(\sigma_{t}^{2}+\sigma_{s}^{2})+\sigma_{e}^{2}}.$$

### The genetic correlation between litters of different size

As already mentioned above the genetic variance of a litter from two parents randomly chosen from the base population equals $n_{i}^{2}(\sigma_{t}^{2}+\sigma_{s}^{2})$, given the number of offspring is $n_{i}$. The genetic covariance of two litters with size $n_{i}$ and $n_{j}$ then is $n_{i}n_{j}(\sigma_{t}^{2}+\sigma_{s}^{2})$ and the genetic correlation between the two litters becomes


$$r_{i,j}=\frac{n_{i}n_{j}(\sigma_{t}^{2}+\sigma_{s}^{2})}{\sqrt{n_{i}^{2}(\sigma_{t}^{2}+\sigma_{s}^{2})n_{j}^{2}(\sigma_{t}^{2}+\sigma_{s}^{2})}}=1.$$

It should be noted that this is different from random regression models for growth traits or milk traits, where the genetic correlation between phenotypes changes over the trajectory of age or lactation (Jamrozik and Schaeffer 1997).

### Models for analysis

The univariate random regression model as applied to the mouse data can be written as


$$y_{ij}=\alpha_{j}+\sum_{k=1}^{2}\phi_{tk}\beta_{k}+x_{i}\beta_{0}+x_{i}u_{m}+x_{i}u_{s}+e_{ij},$$

where $y_{ij}$ is the total LW of the *i*th litter in generation *j* and $\alpha_{j}$is the fixed effect of the *j*th generation. $\phi_{tk}$ are the coefficients of the linear and quadratic (*k* = 1, 2) Legendre polynomials at mating age *t* of the dam and $\beta_{k}$ represents the *k*th regression coefficient of the fixed mating age curve. $x_{i}$ is the transformed LS, where $x_{i}=\frac{n_{i}}{n_{\max}},\;0<x_{i}\leq 1$, and $\beta_{0}$ is the corresponding fixed regression coefficient. $u_{m}$ and $u_{s}$ represent the random genetic effects of the mother *m* and the sire *s* of the litter *i*, respectively, and $e_{ij}$is the residual.

Covariance structures for random effects were specified as: var(u)=G⊗A, and $\mathrm{var}(e)=I_{n}\sigma_{e}^{2}$, where ⊗ denotes the Kronecker product, ***A*** is the numerator relationship matrix, *I_n_* is an identity matrix of order *n* (number of observations), and ***G*** and $\sigma_{e}^{2}$ are defined as described earlier. The fixed generation effect also accounts for the steady increase in inbreeding from one generation to the next. A permanent environment effect was not part of the model due to the absence of repeated litters. The random regression serves a single purpose, which is to multiply the average genetic effects of the dam and the sire by the number of pups. That is why there are no additional random intercepts or random quadratic effects. Results from univariate estimations (with and without $u_{s}$) were compared by a REML log-likelihood ratio test (RLRT; Self and Liang 1987) to test the null hypothesis of a zero sire genetic variance component (trait is completely under maternal control), with 2 degrees of freedom and an error probability of 0.05 as a significance threshold.

Furthermore, we employed bivariate models, where $y_{ij1}$ is the total LW and $y_{ij2}$ is the LS of the *i^th^* litter in generation *j*. Here, random terms $x_{i}u_{m}$ and $x_{i}u_{s}$ were replaced by $u_{m}$ for LS.

Additionally, for the sake of comparison with previous approaches, three different bivariate maternal models for LW and LS were fitted, differing in the treatment of total LW: first, total LW with no adjustment for LS (TLW), second, including a fixed linear regression on LS (LWA), and third, transforming total LW into ABW by dividing total LW by LS (ABW).

For data preparation, we used the R-packages “optiSel” (Wellmann 2019) and self-written programs in R version 4.2.2. (R Core Team 2021). Own R programs were used for calculating functions of genetic parameters, as well as for testing the significance of the sire genetic variance component. Univariate and bivariate REML-estimations of (co)variance components were performed with the help of Echidna MMS software package v1.52 (Gilmour 2018).

## Results

### Litter weight, litter size, and average birth weight

**Figure 2:**
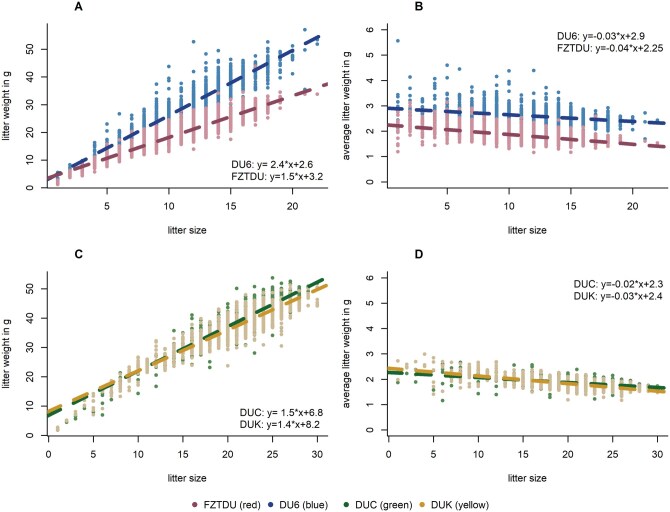
The upper panels show scatter plots of litter weight vs. litter size (A, B). Below is the decline of average birth weight per litter with litter size (C, D). Data for the FZTDU control line and the heavy DU6 line are on the left, while both high fecundity lines DUC and DUK are on the right.

The control line FZTDU had the lowest LW (21 g) among all lines (Table 2). In comparison, the DU6 line, selected on body weight, was about 8 g heavier per litter (∼29 g). While both lines showed almost the same LS, around 11 pups, ABW was considerably higher in DU6 mice, at 2.62 g, compared to 1.81 g in FZTDU controls. The large LW in both high fecundity lines, DUC and DUK, approached as much as 40 g on average. This resulted from their extraordinarily high average LS of about 21, almost equal to the maximum observed LS (22 pups) in FZTDU and DU6. At the same time, ABW in the control (1.81 g) and high fecundity lines (1.84 g in DUC and 1.80 g in DUK) were remarkably similar (Table 2). Thus, in the selection lines, LW has been increased by alternatively modifying either ABV (DU6) or LS (DUC and DUK), while the other component of LS has remained at the level of the controls. Scatter plots of LW vs. LS for all lines in Figure 2 make visible the confounding of LW and LS as a linear trend. Linear regression slopes of LW on LS can be interpreted as the ABW. However, the relationship between LW and LS is not perfectly linear, as the ABW exhibits a certain decline with increasing LS. This effect was later picked up in the negative genetic correlations between the genetic dam effect on LW and breeding values for LS (see below).

### Significance of the sire genetic component

The significance of the sire genetic variance component for LW was investigated by contrasting a full model including a random genetic sire effect and a reduced model without such a component. The test statistic used was the difference between the logarithms of the restricted maximum likelihoods (REML log-likelihood; Table 3). The same test was also applied after transforming LW into ABW and analyzing it traditionally.

**Table 3:** REML log-likelihoods (RL) for the pair-wise comparison of full and reduced models, including (full) and excluding (red) a sire additive-genetic variance, respectively.

Mouse line	Trait	RL_red_	RL_full_	RLRT-value	*P*-value
**FZTDU**	RRM	−4 969.80	−4 961.12	17.36	1.70 ×10^-04^
	ABW	5 427.80	5 429.58	3.56	0.169
**DU6**	RRM	−2 931.68	−2 915.90	31.56	1.40 ×10^-07^
	ABW	1 479.54	1 480.40	1.72	0.42
**DUC**	RRM	−2 898.43	−2 896.78	3.3	0.19
	ABW	3 246.15	3 248.00	1.48	0.47
**DUK**	RRM	−2 884.23	−2 883.43	1.6	0.45
	ABW	2 780.40	2 782.43	4.06	0.13

Test statistics (RLRT) and *P*-values for the test of the null hypothesis of no significant sire genetic variance component are given in the last two columns. The newly proposed random regression model was fitted to total litter weight (RRM). For comparison, LW was transformed to average birth weight per litter (ABW) and modeled traditionally, except for adding a sire effect. Tests were performed for each model for data from the mouse lines FZTDU, DU6, DUC, and DUK.

Given the substantial contribution of the dam variance, omitting the sire effect from the RRM had a small impact on estimates of the residual variance and dam variance. However, when the RRM was used, the genetic sire variance component was highly significant (*P*-values <0.001) in the FZTDU and DU6 lines, but not in the two high-fecundity lines. The pattern of test statistics for the traditional ABW models was quite different, with no significant sire genetic variance observed in any of the lines (Table 3).

### Genetic parameters

**Figure 3:**
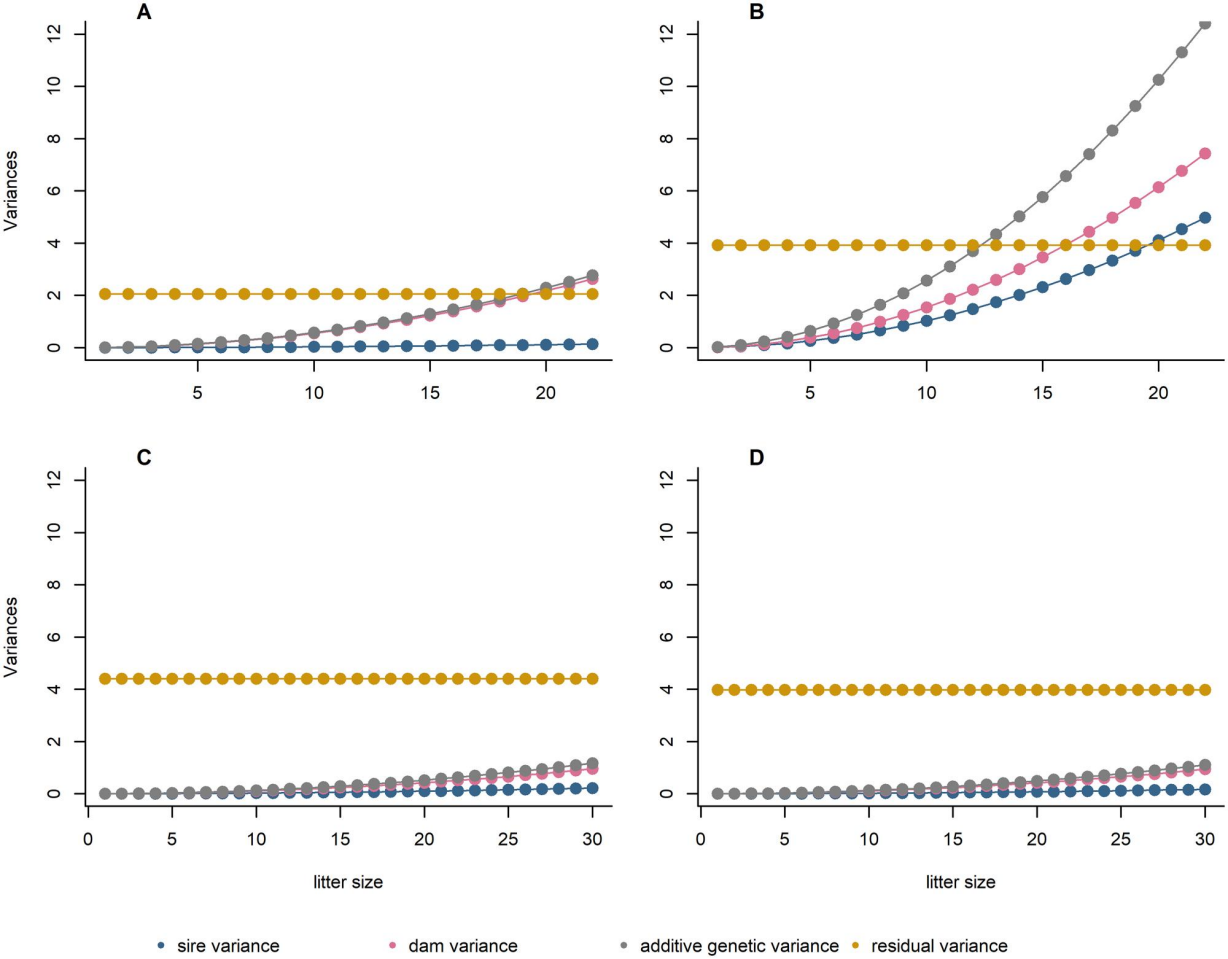
Genetic variances for litter weight for each litter size of the mouse lines FZTDU (A), DU6 (B), DUC (C), and DUK (D).

Estimates of genetic and residual (co-)variance components are shown in Table 4. Due to our chosen scaling, the genetic (co-)variances are valid at the maximum LS and *x_i_* = 1, which is at 22 pups in FZTDU and DU6, and 30 pups in DUC and DUK. Estimates of the sire variance at maximum LS in FZTDU, DUC, and DUK were of a similar magnitude, whereas in DU6 the sire component was about 25-fold larger (Table 4). In terms of its relative size, the sire genetic variance was also the highest in the growth-selected DU6 line at 67% of the estimate for the dam variance. In the two high fecundity lines DUC and DUK, the respective ratios were at 23% and 18%, and in the unselected control line FZTDU, the sire variance was only 5% of the dam variance. When compared at equal LS (Figure 3), estimated absolute values of the total additive genetic variance as well as its components were highest in DU6, followed by FZTDU, and considerably smaller in DUC and DUK. In particular, sire genetic variances in the latter two lines were the least at about 72% and 55% of the sire variance in FZTDU, when compared at equal LS. The latter is in line with the aforementioned non-significance of the sire component in DUC and DUK. While in pups of DU6 and FZTDU, the total genetic variance and at least one of its components exceeded the residual variance at higher LS, this was not the case at all in DUC and DUK, where the genetic variance was at a considerably lower level (Figure 3). The residual variances in the selection lines DU6, DUC, and DUK were all around four, compared to only half of that in the unselected control line FZTDU (Table 4; Figure 3), adding to the significance of the sire component in the latter line. No obvious increase in the residual variance with LS became apparent by visual inspection of residual plots (data not shown). Genetic correlations between dam and sire effects were estimated as positive and ranged from 0.398 to 0.993, with the only moderately negative estimate at −0.359 in DU6 (Table 4). Standard errors for the genetic variance components, as given in Table 4, apply to the maximum LS. Standard errors of genetic variances for any other LS, as displayed in Figure 3, can easily be obtained by multiplying by the respective standardized LS $x_{i}$.

**Table 4:** Estimates of (co-)variance components of litter weight in the mouse lines FZTDU, DU6, DUC, and DUK, as estimated with the random regression model.

	Parameter				
Lines	$\sigma_{t}^{2}$	$\sigma_{s}^{2}$	$\sigma_{t,s}$	r_ts_	$\sigma_{e}^{2}$
**FZTDU**	2.631 (±0.053)	0.137 (±0.003)	0.596 (±0.012)	0.99 (±0.00)	2.056 (±0.042)
**DU6**	7.433 (±1.247)	4.976 (±1.051)	−2.181 (±0.940)	−0.36 (±.14)	3.925 (±0.283)
**DUC**	0.949 (±0.029)	0.220 (±0.007)	0.406 (±0.012)	0.89 (±0.00)	4.408 (±0.134)
**DUK**	0.937 (±0.028)	0.168 (±0.005)	0.158 (±0.007 0.005)	0.41 (±0.01)	3.976 (±0.119)

Standard errors in brackets. Standard errors for the total genetic variance $\sigma_{a}^{2}$ were 0.056, 1.520, 0.036, and 0.033, respectively.

Abbreviations: $\sigma_{a}^{2}=\sigma_{t}^{2}+\sigma_{s}^{2}$, total genetic variance; $\sigma_{t}^{2}$=, genetic variance component of the dam; $\sigma_{s}^{2}$, genetic variance component of the sire; $\sigma_{t,s}$, genetic covariance component between the dam and the sire; $r_{ts}$, genetic correlation between the genetic dam and sire effects; $\sigma_{e}^{2}$, residual variance.

**Figure 4:**
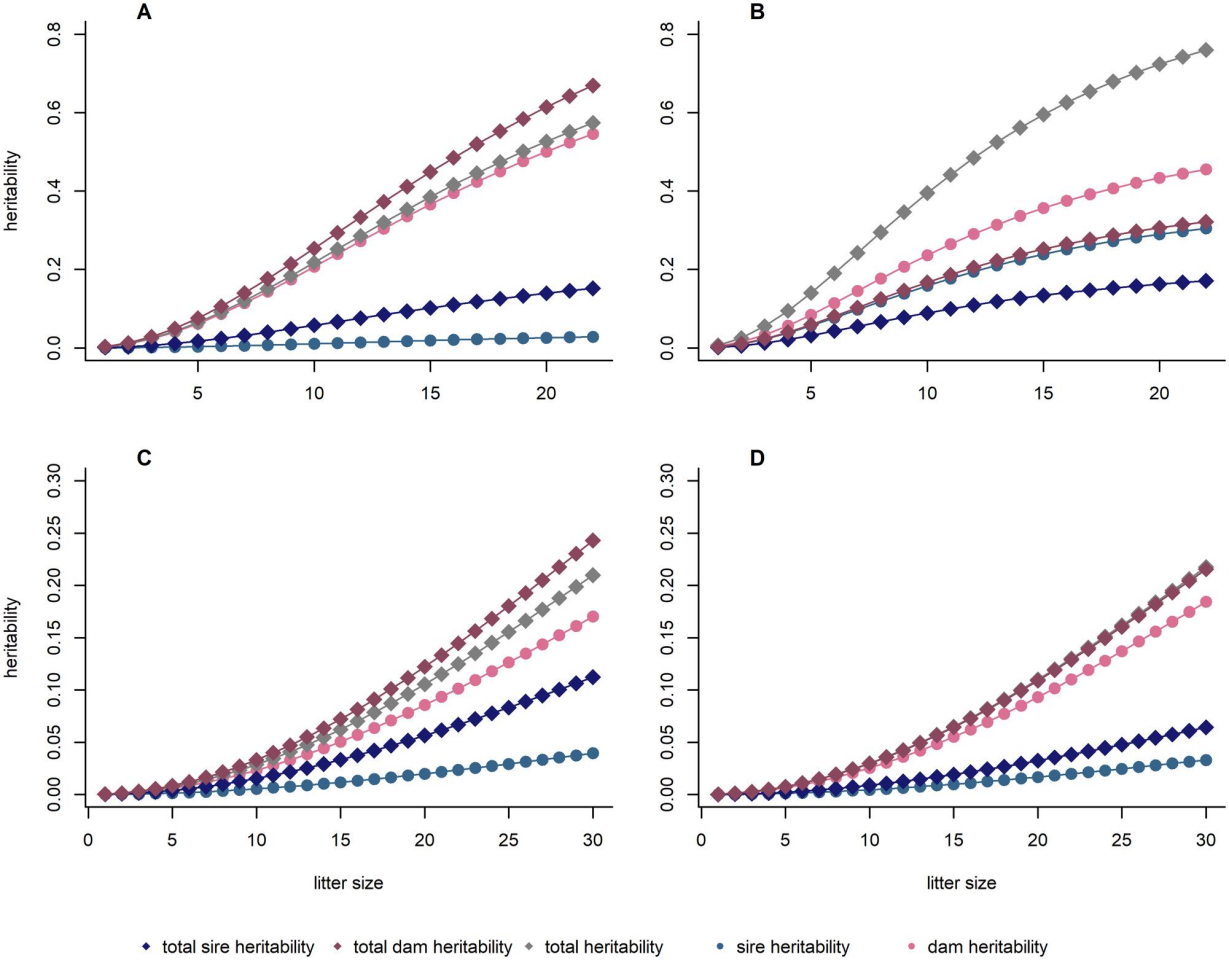
Heritability estimates for litter weight for each litter size of the mouse lines FZTDU (A), DU6 (B), DUC (C), and DUK (D).

Estimates for average heritability are presented in Table 5 for all lines, where averaging was over line-specific distributions of LS. The average dam heritability was considerably higher at 26% in FZTDU and DU6, compared to 10% in the high fecundity lines DUC and DUK. The average sire heritability was very small at 1% to 2% in all lines except DU6, with 17%. Consequently, the average total heritability did not markedly deviate from the dam heritability in FZTDU, DUC, and DUK. In the DU6 line, however, total heritability was considerably higher at 47%, both when compared with the dam heritability in the same line and compared with total heritability in all other lines. Trajectories of heritability estimates over the line-specific ranges of LS are shown in Figure 4. Corresponding information on standard errors can be found in the Supplementary Appendix. Total heritability in FZTDU and DU6 peaked at about 60% when LS reached its maximum of 22 pups. Total heritability was by far smaller at the same LS level in DUC and DUK and hardly exceeded 20%, even at an LS of 30. For comparison, heritability estimates for ABW from a conventional model are shown in Table 6. Estimates ranged between 8.7% and 20.3%, and only in DUC did the estimate exceed that for average total heritability from the RRM. Further, estimates for the heritability for LS were quite different from what was observed for LW (Table 6), all in a narrow range between 13.5% and 16.9%, except for DUK, where the estimate was only 4.5%.

**Table 5:** Estimates for average dam heritability($h_{d}^{2}$), sire heritability ($h_{s}^{2}$), and total heritability ($h_{\mathrm{tot}}^{2}$) of litter weight in mouse lines FZTDU, DU6, DUC, and DUK, as derived from the random regression model.

Lines	$h_{d}^{2}$	$h_{s}^{2}$	$h_{\mathrm{tot}}^{2}$
**FZTDU**	0.26 (±0.00)	0.01 (±0.00)	0.27 (±0.00)
**DU6**	0.26 (±0.03)	0.17 (±0.03)	0.43 (±0.05)
**DUC**	0.10 (±0.00)	0.02 (±0.00)	0.12 (±0.00)
**DUK**	0.10 (±0.00)	0.02 (±0.00)	0.12 (±0.00)

Standard errors in brackets.

**Table 6:** Genetic variance, residual variance, and heritability for the traits litter size and average birthweight in the mouse lines FZTDU, DU6, DUC, and DUK.

		Trait	
Mouse line	Parameter	Litter size	Average birth weight
**FZTDU**	$\sigma_{a}^{2}$	1.218 (±0.223)	0.0079 (±0.001)
	$\sigma_{e}^{2}$	7.758 (±0.243)	0.031 (±0.001)
	$h_{}^{2}$	0.135 (±0.033)	0.203 (±0.026)
**DU6**	$\sigma_{a}^{2}$	2.238 (±0.569)	0.0067 (±0.003)
	$\sigma_{e}^{2}$	10.949 (±0.532)	0.0701 (±0.003)
	$h_{}^{2}$	0.169 (±0.041)	0.087 (±0.0035)
**DUC**	$\sigma_{a}^{2}$	1.598 (±0.476)	0.0043 (±0.0008)
	$\sigma_{e}^{2}$	9.598 (±0.466)	0.0135 (±0.0007)
	$h_{}^{2}$	0.143 (±0.041)	0.242 (±0.044)
**DUK**	$\sigma_{a}^{2}$	0.738 (±0.569)	0.0026 (±0.0011)
	$\sigma_{e}^{2}$	15.717 (±0.677)	0.0261 (±0.0012)
	$h_{}^{2}$	0.045 (±0.034)	0.091 (±0.037)

Standard errors in brackets.

Abbreviations: $\sigma_{a}^{2}$, genetic variance; $\sigma_{e}^{2}$, residual variance; $h_{}^{2}$, heritability.

Estimates for the genetic correlation between the genetic dam effect on LW as defined in the RRM and the dam’s breeding value for LS were uniformly negative (Table 7) across all lines, albeit varying between -0.37 and -0.92. In contrast, when LW was modeled in the traditional ways, estimates for the respective genetic correlations with LS were at a very high positive level in all lines, with values ranging from 0.78 to 0.98, no matter of whether LW was analyzed with (LWA) or without (LW) linear adjustment for LS (Table 7). No such uniform pattern could be observed when the third version of a traditional LW model was applied by using ABW, and estimates were considerably lower.

**Table 7:** Estimated genetic correlations between the traits litter size (LS) and total litter weight in the mouse lines FZTDU, DU6, DUC, and DUK.

	Mouse line			
Trait	FZTDU	DU6	DUC	DUK
**LS-RRM**	−0.37 (±0.07)	−0.51 (±0.10)	−0.67 (±0.12)	−0.92 (±0.11)
**LS-LW**	0.89 (±0.02)	0.98 (±0.01)	0.85 (±0.05)	0.89 (±0.06)
**LS-LWA**	0.89 (±0.02)	0.93 (±0.03)	0.85 (±0.05)	0.78 (±0.11)
**LS-ABW**	−0.23 (±0.10)	0.19 (±0.27)	−0.47 (±0.13)	−0.20 (±0.38)

Four different model variants for litter weight were used: unadjusted (LW), adjusted (LWA), random regression model (RRM), and averaged litter weights (ABW). Standard errors in brackets.

### Reliabilities

**Figure 5:**
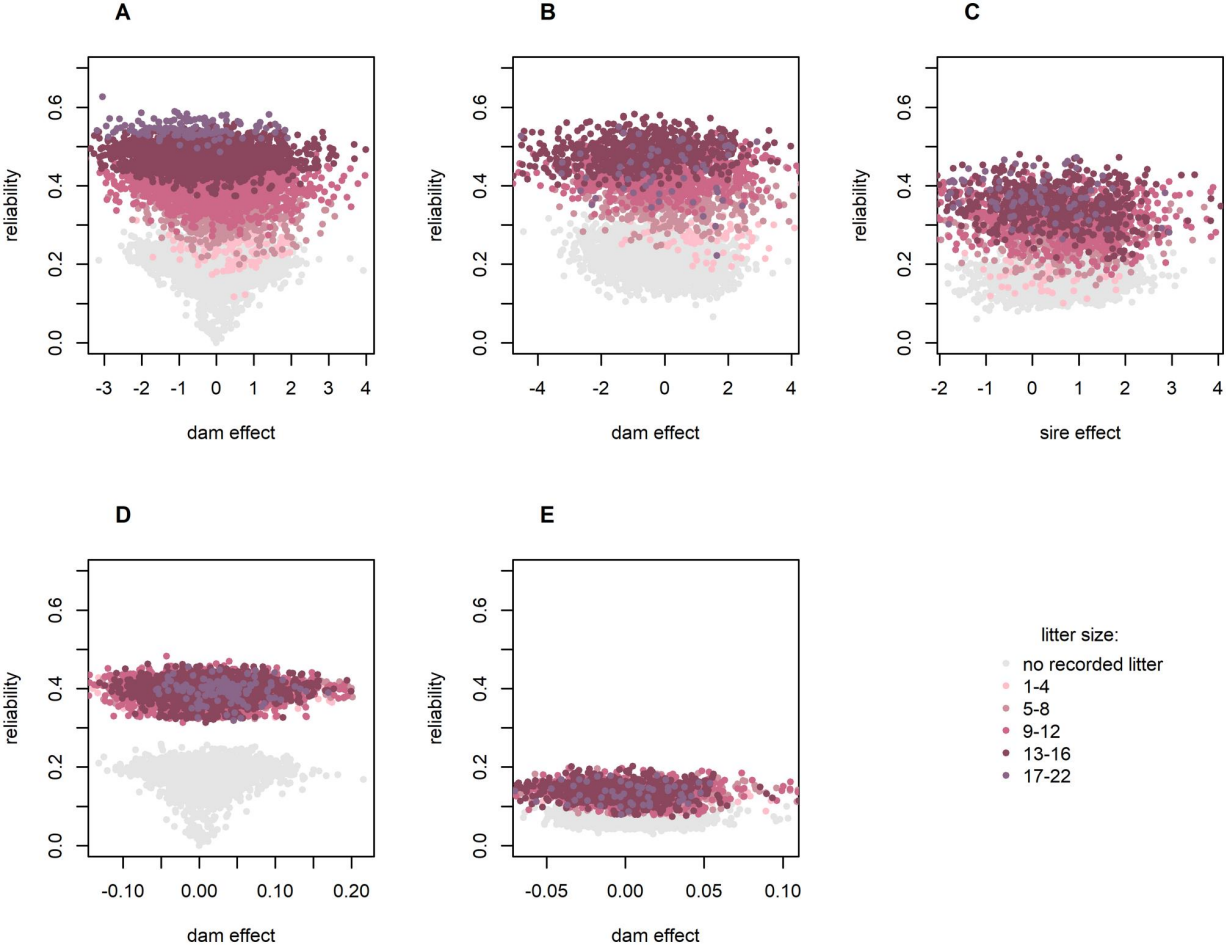
Plots of the reliability of estimates vs. their size. Top: Reliabilities from the random regression model for litter weight for dam effects in FZTDU (A) and DU6 (B) mice and for the sire effect in the DU6 line (C). Bottom: Reliabilities for dam effects from a conventional model for average litter weight in the lines FZTDU (D) and DU6 (E). Colored layers indicating litter size visualize that the reliability of estimates increases with larger litters in the random regression model (top). The absence of this effect in the conventional model for average litter weight becomes apparent by the random and overlapping positions of the colored data points in a single layer (bottom).

In the RRM, reliabilities for the dam effects increased for animals with higher LS, shown in Figure 5A and B, where a higher LS is indicated by darker reddish dots. In the FZTDU line, the reliability of the estimated dam effects for LW reached 63% (Figure 5A) and was almost identical in DU6 with 61% (Figure 5B). In contrast, when LW was analyzed in the traditional way as ABW, reliabilities were independent of LS. Here, the highest reliabilities reached up to 48% (Figure 5C) in FZTDU, and up to 20% in DU6 (Figure 5D). That difference is a consequence of the heritability increasing with LS in the random regression model, while heritability in the traditional ABW model does not depend on LS.

## Discussion

Results from the RRM considerably differ from traditional models for LW with respect to three major aspects: the amount of genetic variation—including the sire component—of LW, the genetic covariation with LS, and, finally, the effect of LS on the heritability of LW and, hence, on the reliability of estimated genetic effects. All these differences originate from the RRM’s ability to overcome the confounding of LW with LS and effectively extract the information on the ABW of pups, as one of the two component traits of LW.

The largest sire genetic variance component was found in the heavy DU6 line. In contrast, the same component was comparatively smaller in FZTDU at comparable LS, and even smaller and non-significant in DUC and DUK. For FZTDU, the sire component being smaller than in DU6 may appear counterintuitive, as FZTDU has previously neither been selected for body weight nor any other trait, nor has there been an opportunity for losing much of its genetic variability, as the line has been maintained at a considerably higher population size before and after the aforementioned bottleneck. Comparative DNA-sequence data have shown a much higher level of overall heterozygosity in FZTDU than in DU6, DUC, and DUK (Palma-Vera et al. 2022), in good agreement with pedigree information. Presumably, genetic variability in the DU6 line has been scaled up by a strongly correlated selection response for birth weight, a consequence of the long-term selection on body weight at 42 d of age, which, on average, has been elevated to more than two times that in control mice. There is a connected change of variance components. Another aspect is the family structure in DU6, where 13.9% of all bucks sired two litters, which is likely somewhat better suited for a sound partitioning of all (co-) variance components, compared to the other lines, where bucks had not more than a single litter.

The significance of the sire genetic variance in two lines is in contrast to the traditional ABW model, where the sire variance was not significant in any of the four lines. Previous genetic analyses, to the best of our knowledge, have considered LW as a trait of the dam, neglecting sire effects on LW traits. Especially the DU6 example, however, demonstrates that a genetic sire effect on total LW is detectable by statistical means. As the sire variance stands for a quarter of the direct genetic variation of birth weight, the total absence of such a variance component is not plausible. The fact that with other trait definitions, especially ABW, the sire genetic variance could not be detected in any of the lines, questions the usefulness and validity of the traditional definitions and models for LW. On the other hand, the proposed RRM is in full compliance with a direct-maternal animal model for individual birth weight.

The genetic correlations between LW and LS from the RRM were estimated as negative in all lines (Table 4). These negative estimates were in agreement with various studies of individual birth weight in pigs where genetic correlations between that trait and LS ranged between -0.72 and -0.18 (Damgaard et al. 2003; Kapell et al. 2011; Sell-Kubiak 2021; Zaalberg et al. 2023). Other studies, considering LW a trait of the dam without any adjustment for LS, found genetic correlations between LW and LS between 0.85 and 0.96 (Gutiérrez et al. 2006; Luxford and Beilharz 1990; Ogawa and Satoh 2020), very well in agreement with estimates from our data when the same kind of analyses was applied (Table 4). Only Schüler et al. (1990) found a somewhat lower, yet still markedly positive, genetic correlation between these traits of 0.59. Thus, unadjusted LW genetically is more of a proxy for LS, just expressed in units of weight rather than as the number of pups. Adding LS as a covariate solely in the fixed part of the model led to almost no changes (Table 4). In contrast, the estimated genetic correlations between ABW and LS were much smaller, with no uniform pattern of signs in our study. Estimates were comparable in size with those in Ogawa and Satoh (2020), yet lower than the negative estimate of −0.65 reported by Gutiérrez et al. (2006).

Estimates of the average heritability in the RRM were not strikingly different from heritability estimates for LW obtained with traditional models in previous studies. While Gutiérrez et al. (2006) reported a heritability of 0.11, assuming maternal genetic effects, Luxford and Beilharz (1990) found a heritability of 0.24 in LW. A recent study (Ogawa and Satoh 2020) found a heritability for LW of 0.39 ± 0.09 in an unselected mouse line, in good agreement with the average heritability of LW in FZTDU. Additionally, for ABW, a similar heritability of 0.24 was described (Ogawa and Satoh 2020). However, heritability changing with LS is a new feature that is alien to all the traditional models. As a logical consequence and as visually demonstrated in Figure 5, the reliability of the estimated genetic effects changes with LS in the RRM according to the varying amount of information on ABW in small or large litters. Traditional models, in contrast, treat litters of all sizes alike, ignoring any related effects on precision.

In summary, we conclude that a random regression model that uses LS as a covariate and includes parental genetic effects as random linear slope effects makes more efficient use of the available data in focusing on the birth weight component of the trait. The RRM also has the advantage that it accounts for the variable amount of information that litters of different size provide for the estimation of genetic effects. When applied to other species or data sets, the model may need specific adaptations like the inclusion of permanent environment effects or heterogeneous residual variances. In general, however, the treatment of LW in the random regression model follows more the well-established understanding of the genetics of individual birth weight, for which a combination of maternal and direct genetic effects is commonplace in the literature. Therefore, the random regression model may find adapted applications in the breeding of other multiparous species in the future, especially when the recording of individual birth weight on a larger number of farms is too costly.

## Conclusion

In this study, we introduce a random regression model for total LW in mice. Genetic variance and heritability are treated as a function of observed LS. Further, the model easily allows for a genetic sire effect. Potential applications are in genetic evaluations in all kinds of multiparous species when only total LW is recorded instead of individual birth weight.

## Supplementary Material

skag060_Supplementary_Data

## Abbreviations

ABW: average birth weight
LS: litter size
LW: litter weight
LWA: litter weight adjusted to litter size
TLW: total litter weight, no adjustment for litter size
RLRT: REML log-likelihood ratio test
RRM: random regression model for LW
