# Supplementary material for: A random regression model for total litter weight in mice
Source: J Anim Sci. 2026 Feb 23;104:skag060. doi: 10.1093/jas/skag060 (PMC13242930; doi:10.1093/jas/skag060)
Supplement: skag060_Supplementary_Data [file skag060_supplementary_data.docx]

**Appendix: Standard errors of heritability estimates**

Computing the standard errors of heritability estimates at each specific LS involves the sampling error covariance matrix of covariance parameters (inverse of the average information matrix):

$Var\left[\begin{matrix} \sigma_{e}^{2} & \sigma_{t}^{2} & \sigma_{t,s} & \sigma_{s}^{2} \end{matrix}\right]^{'}=\mathbf{V}_{vc}$.

Estimates of these four-by-four matrices are given in Table **A1** for all lines.

**Table A1:** Sampling error covariance matrices of the genetic covariance components (dam variance, covariance between dam and sire effects, sire variance) and residual variance for lines FZTDU, DU6, DUC, and DUK (clockwise from upper left).

| | 0.0017389 | 0.002225 | 0.000504 | 0.0001157 | | --- | --- | --- | --- | | 0.002225 | 0.0028469 | 0.0006449 | 0.000148 | | 0.000504 | 0.0006449 | 0.0001461 | 0.0000335 | | 0.0001157 | 0.000148 | 0.0000335 | 7.6983E-6 | | | 0.0800449 | -0.189803 | 0.0955731 | -0.133111 | | --- | --- | --- | --- | | -0.189803 | 1.55549 | -0.336955 | -0.174725 | | 0.0955731 | -0.336955 | 0.883822 | -0.227909 | | -0.133111 | -0.174725 | -0.227909 | 1.10426 | |
| --- | --- | --- | --- | --- | --- | --- | --- | --- | --- | --- | --- | --- | --- | --- | --- | --- | --- | --- | --- | --- | --- | --- | --- | --- | --- | --- | --- | --- | --- | --- | --- | --- | --- |
| | 0.0179467 | 0.0038655 | 0.0016518 | 0.0008966 | | --- | --- | --- | --- | | 0.0038655 | 0.0008326 | 0.0003558 | 0.0001931 | | 0.0016518 | 0.0003558 | 0.000152 | 0.0000825 | | 0.0008966 | 0.0001931 | 0.0000825 | 0.0000448 | | | 0.0141065 | 0.0033235 | 0.0005603 | 0.000597 | | --- | --- | --- | --- | | 0.0033235 | 0.000783 | 0.000132 | 0.0001407 | | 0.0005603 | 0.000132 | 0.0000223 | 0.0000237 | | 0.000597 | 0.0001407 | 0.0000237 | 0.0000253 | |

0.173890E-02

0.222498E-02 0.284694E-02

0.504013E-03 0.644903E-03 0.146087E-03

0.115700E-03 0.148042E-03 0.335352E-04 0.769825E-05

For each type of heritability estimate, a two-by-two sampling covariance matrix$\mathbf{V}$can be drawn from$\mathbf{V}_{vc}$ by the transformation $\mathbf{TV}_{vc}\mathbf{T}'$ , which is the sampling covariance matrix of the genetic component in the denominator of the respective estimate and the residual variance. Transformation matrices specific for the dam heritability, sire heritability, and the total heritability are summarized in Table A2.

**Table A2:** Transformation matrices **T** by type of estimate.

| h2 dam | h2 sire | h2 total |
| --- | --- | --- |
| | 0 | 1 | 0 | 0 | | --- | --- | --- | --- | | 1 | 0 | 0 | 0 | | | 0 | 0 | 0 | 1 | | --- | --- | --- | --- | | 1 | 0 | 0 | 0 | | | 0 | 1 | 0 | 1 | | --- | --- | --- | --- | | 1 | 0 | 0 | 0 | |

Partial derivatives of the dam (sire, total) heritability with respect to the dam (sire, total) variance and residual variance were written to the first and second columns of a matrix$\mathbf{M}$with two columns and one row indexed i for each particular LS (e.g., 22 rows in FZTDU and DU6). These partial derivatives were computed as follows.

$$\frac{\partial h_{dam}^{2}}{\partial\sigma_{d}^{2}}=\frac{x_{_{i}}^{4}\sigma_{s}^{2}+x_{_{i}}^{2}\sigma_{e}^{2}}{\left(x_{_{i}}^{2}\sigma_{a}^{2}+\sigma_{e}^{2}\right)^{2}}$$

$$\frac{\partial h_{dam}^{2}}{\partial\sigma_{e}^{2}}=\frac{-x_{i}^{2}\sigma_{d}^{2}}{\left(x_{i}^{2}\sigma_{a}^{2}+\sigma_{e}^{2}\right)^{2}}$$

$$\frac{\partial h_{sire}^{2}}{\partial\sigma_{s}^{2}}=\frac{x_{_{i}}^{4}\sigma_{dam}^{2}+x_{_{i}}^{2}\sigma_{e}^{2}}{\left(x_{_{i}}^{2}\sigma_{a}^{2}+\sigma_{e}^{2}\right)^{2}}$$

$$\frac{\partial h_{sire}^{2}}{\partial\sigma_{e}^{2}}=\frac{-x_{i}^{2}\sigma_{s}^{2}}{\left(x_{i}^{2}\sigma_{a}^{2}+\sigma_{e}^{2}\right)^{2}}$$

$$\frac{\partial h_{tot}^{2}}{\partial\sigma_{a}^{2}}=\frac{x_{_{i}}^{2}\sigma_{e}^{2}}{\left(x_{_{i}}^{2}\sigma_{a}^{2}+\sigma_{e}^{2}\right)^{2}}$$

$$\frac{\partial h_{tot}^{2}}{\partial\sigma_{e}^{2}}=\frac{-n_{i}^{2}\sigma_{a}^{2}}{\left(n_{i}^{2}\sigma_{a}^{2}+\sigma_{e}^{2}\right)^{2}}$$

Then the matrix product $\mathbf{MV}\mathbf{M}'$results in a matrix with sampling error variances for each LS on the diagonal.

Finally, the sampling error variance of the average heritability is given by

$Var\left(\mathbf{f}'\mathbf{h}\right)=Var\left(h_{average}^{2}\right)=\mathbf{f}'\left(\mathbf{MV}\mathbf{M}'\right)\mathbf{f}$,

where$\mathbf{f}$is a column vector with line-specific relative frequencies of litters with different LS.

**Table A3:** Maximum standard errors for LS-specific heritability estimates by line and type of estimate.

|  | h2 dam | h2 sire | h2 total | max |
| --- | --- | --- | --- | --- |
| FZTDU | 0.000027466 to 0.0040506 | 1.4351974E-6 to  0.000507972 | 6.8014935E-8 to  6.1479748E-6 | 0.004 |
| DU6 | 0.000690223 to  0.0418690 | 0.000570845 to  0.0448823 | 0.0014373 to  0.0556769 | 0.06 |
| DUC | 5.6212017E-6 to 0.0035599 | .3041534E-6 to  0.000982615 | 0.00 to 0.00 | 0.004 |
| DUK | 5.8506993E-6 to  0.0036440 | 1.0512859E-6 to 0.000804213 | 1.1487416E-8 to  6.3346751E-6 | 0.004 |

FZTDU sterrors:

| 0.0417001 | 0.0533567 | 0.0120866 | 0.0027746 |
| --- | --- | --- | --- |

Total: 0.0561313

Sterr average s2a: 4.6703E-6 s2d: 0.0024909 s2d: 0.0002054

DU6 standard errors:

| 0.2829221 | 1.2471928 | 0.9401181 | 1.0508378 |
| --- | --- | --- | --- |

Total: 1.5199671

Sterr average s2a: 0.0490374 s2d: 0.0312384 s2s: 0.029607

DUC standard errors:

| 0.1339653 | 0.0288548 | 0.01233 | 0.0066929 |
| --- | --- | --- | --- |

Total: 0.0355477

Sterr average s2a: 0.00 s2d:0.0022072 s2s: 0.00057

**DUK**

Standard errors:

| 0.1187708 | 0.0279824 | 0.0047179 | 0.0050266 |
| --- | --- | --- | --- |

Total: 0.033009

Sterr average s2a: 3.9445E-6 s2d: 0.0021606 s2s: 0.0004396
